# Distinct pattern of genomic breakpoints in CML and *BCR::ABL1*-positive ALL: analysis of 971 patients

**DOI:** 10.1186/s12943-024-02053-4

**Published:** 2024-07-05

**Authors:** Lenka Hovorkova, Lucie Winkowska, Justina Skorepova, Manuela Krumbholz, Adela Benesova, Vaclava Polivkova, Julia Alten, Michela Bardini, Claus Meyer, Rathana Kim, Toby N. Trahair, Emmanuelle Clappier, Sabina Chiaretti, Michelle Henderson, Rosemary Sutton, Lucie Sramkova, Jan Stary, Katerina Machova Polakova, Rolf Marschalek, Markus Metzler, Giovanni Cazzaniga, Gunnar Cario, Jan Trka, Marketa Zaliova, Jan Zuna

**Affiliations:** 1 CLIP – Childhood Leukaemia Investigation Prague, Prague, Czech Republic; 2 Department of Paediatric Haematology and Oncology, Second Faculty of Medicine, Charles University/University Hospital Motol, Prague, Czech Republic; 3 Pediatric Oncology and Hematology, Department of Pediatrics and Adolescent Medicine, University Hospital, Erlangen, Germany; 4 Institute of Hematology and Blood Transfusion, Prague, Czech Republic; 5 Department of Pediatrics, University Hospital Schleswig-Holstein, Kiel, Germany; 6 Tettamanti Center, Fondazione IRCCS San Gerardo dei Tintori, Monza, Italy; 7 Institute of Pharmaceutical Biology/Diagnostic Center of Acute Leukemia, Goethe-University, Frankfurt, Germany; 8 Hematology laboratory, AP-HP, Saint-Louis hospital, Université Paris-Cité, Paris, France; 9 Children’s Cancer Institute, Randwick, Australia; 10 School of Women’s and Children’s Health, School of Medicine, University of NSW, Sydney, Australia; 11 Kids Cancer Centre, Sydney Children’s Hospital Randwick, Sydney, Australia; 12 Division of Hematology, Department of Translational and Precision Medicine, Sapienza University of Rome, Rome, Italy; 13 Medical Genetics, School of Medicine and Surgery, Univ. Milano Bicocca, Monza, Italy

**Keywords:** Acute lymphoblastic leukemia, Chronic myeloid leukemia, Genomic breakpoints, BCR, ABL1

## Abstract

**Background:**

The *BCR::ABL1* is a hallmark of chronic myeloid leukemia (CML) and is also found in acute lymphoblastic leukemia (ALL). Most genomic breaks on the *BCR* side occur in two regions - Major and minor - leading to p210 and p190 fusion proteins, respectively.

**Methods:**

By multiplex long-distance PCR or next-generation sequencing technology we characterized the *BCR::ABL1* genomic fusion in 971 patients (adults and children, with CML and ALL: pediatric ALL: *n* = 353; pediatric CML: *n* = 197; adult ALL: *n* = 166; adult CML: *n* = 255 patients) and designed “Break-App” web tool to allow visualization and various analyses of the breakpoints. Pearson’s Chi-Squared test, Kolmogorov-Smirnov test and logistic regression were used for statistical analyses.

**Results:**

Detailed analysis showed a non-random distribution of breaks in both *BCR* regions, whereas *ABL1* breaks were distributed more evenly. However, we found a significant difference in the distribution of breaks between CML and ALL. We found no association of breakpoints with any type of interspersed repeats or DNA motifs. With a few exceptions, the primary structure of the fusions suggests non-homologous end joining being responsible for the *BCR* and *ABL1* gene fusions. Analysis of reciprocal *ABL1::BCR* fusions in 453 patients showed mostly balanced translocations without major deletions or duplications.

**Conclusions:**

Taken together, our data suggest that physical colocalization and chromatin accessibility, which change with the developmental stage of the cell (hence the difference between ALL and CML), are more critical factors influencing breakpoint localization than presence of specific DNA motifs.

**Supplementary Information:**

The online version contains supplementary material available at 10.1186/s12943-024-02053-4.

The *BCR::ABL1* fusion gene is not only a hallmark of chronic myeloid leukemia (CML) but is also present in a proportion of patients (3–5% in children, 20–30% in adults) with acute lymphoblastic leukemia (ALL). The t(9;22)(q34;q11) translocation, recognized at the chromosomal level as “Philadelphia chromosome”, involves DNA breaks in large, mostly intronic regions of the *BCR* and *ABL1* genes. The majority of breakpoints on the *BCR* side occur in two “breakpoint cluster regions” – “minor” (between exons 1 and 2; ~ 71.5 kilobase pairs [kbp], resulting in the p190 fusion protein, prevalent in ALL and scarce in CML) and “Major” (between exons 13 and 15; ~ 2.9 kbp, resulting in the p210 fusion protein, less frequent in ALL and almost exclusive in CML). On the *ABL1* side, breakpoints are mostly localized between exon 1 and exon 2 (~ 140 kbp), however, breaks upstream of the *ABL1* have also been described [1, 2].

Numerous studies have been published focusing on aberrant expression and downstream signaling of the *BCR::ABL1* fusion gene/protein. However, due to the large intronic areas where the breakpoints occur, only a few papers have been published focusing on the primary structure of the *BCR::ABL1* fusions. Moreover, to our knowledge, only one study included (a limited number of) patients with the minor form of *BCR::ABL1* fusion (*n* = 25) and suggested RAG involvement in the double-strand break initiation in a subset of ALL [2]. Analysis focused on the localization of breakpoint sites in the Major *BCR* area showed a bimodal distribution of breakpoints [3, 4]. In contrast, the distribution of breakpoints in *ABL1* was shown to be more uniform; however, some studies suggested subtle differences in distribution with respect to the gender or age of patients [3, 5].

Several analyses concerned the localization of breakpoints within *BCR* and *ABL1* genes with respect to the presence of interspersed repeats (IR), recombination signal sequences for RAG-recombinase (RSS), or motifs known to mediate DNA breaks (e.g. cleavage sites for topoisomerases, immunoglobulin switch sequences, etc.) [1–4]. The primary structure of fusion sequences – mostly represented by short homologies, blunt-end connections or short insertions of random nucleotides – has led to the hypothesis that non-homologous end joining (NHEJ) is probably responsible for the *BCR* and *ABL1* fusion [1, 4, 6].

In the present study, to definitively assess these factors and analyze genomic fusions in an extensive and fully representative cohort, we searched for and identified *BCR::ABL1* genomic breakpoints in 971 patients with *BCR::ABL1-*positive ALL (*n* = 519) and CML (*n* = 452). Our cohort includes both pediatric (*BCR::ABL1*-positive ALL: *n* = 353; CML: *n* = 197) and adult (*BCR::ABL1*-positive ALL: *n* = 166; CML: *n* = 255) patients, with part of the sequences published previously [3, 7–9]. In general, all included patients were sent to the *BCR::ABL1* genomic breakpoint identification after the presence of the fusion was already revealed by routine diagnostics - cytogenetics and/or reverse-transcriptase (RT-) PCR. Hence, our cohort might be slightly negatively biased towards very unusual fusions, missed by routine diagnostics – while our NGS approach with probes covering both *BCR* and *ABL1* breakpoint regions is capable to detect also unusual breakpoints, such cases (e.g. micro-*BCR::ABL1*) might be underrepresented in our study.

The *BCR::ABL1* breakpoints were characterized originally by long-distance PCR (*n* = 427) and later by target enrichment NGS (*n* = 544) with custom-designed probes covering the following areas (according to GRCh38/hg38): minor *BCR* - chr22:23,180,958 − 23,254,000; Major *BCR* - chr22:23,289,491 − 23,292,664; micro *BCR* - chr22:23,311,732 − 23,313,035; (*EXOSC2*)/*ABL1* - chr9:130,699,582 − 130,855,101. While breakpoint identification by PCR yielded some negative results (the proportion of unsuccessful attempts to obtain a genomic breakpoint was approximately 10–15%), the success rate by NGS was close to 100% (with rare failures attributable mainly to poor DNA quality or very low [< 5%] blast percentage). For more details see Additional methods.

Of the theoretically expected 1942 genomic breakpoints in *BCR* and *ABL1* genes, we identified exact position for 1935 breakpoints (396, 575 and 964 breakpoints in the minor *BCR*, Major *BCR* and *ABL1*, respectively). In six patients examined in the early part of the study, the *ABL1* breakpoints were not precisely characterized (three fusions with a breakpoint upstream of *ABL1* and three fusions with a large *ABL1* inversion) and one patient had a repetitive sequence that prevented reliable precise identification of the *ABL1* breakpoint position. The breakpoints were mostly located in introns (*n* = 1852; 96% of all identified breakpoints); however, 40 patients (4%) had genomic breakpoints upstream of *ABL1* (*n* = 8 in *EXOSC2* gene; *n* = 32 in intergenic area between *EXOSC2* and *ABL1*), and 45 patients had breakpoints in exons (*ABL1* exon 1, *n* = 6; *ABL1* exon 1b, *n* = 2; *ABL1* exon 2, *n* = 1; *BCR* exon 2, *n* = 1; *BCR* exon 14, *n* = 25; *BCR* exon 15, *n* = 10). The breakpoint sites covered almost the complete breakpoint areas with only a few gaps larger than 1kbp, where no breakpoint was detected (minor *BCR*: *n* = 8; 1.1–3.3 kbp; *ABL1*: *n* = 7; 1.1–2.3 kbp). Where analyzed, the transcript variant (minor vs. Major) always corresponded to the genomic fusion localization. As already described, in some patients with Major *BCR::ABL1* fusion, low levels of minor *BCR::ABL1* transcript are also expressed due to alternative splicing; only the Major *BCR::ABL1* genomic breakpoint was identified in all such cases. For all breakpoint positions and basic characteristics of the patients see Additional Table 1.

The uniformity test performed on breakpoint distribution within the minor (n = 396) and Major (n = 575) *BCR* areas revealed a non-random pattern (p = 1.89e-22 and p = 1.45e-11, respectively; see Fig. 1A, B) with breakpoints accumulating in the 3’ end area of the intron 1 (minor *BCR*) and in intron 13 (Major *BCR*). Within the *ABL1* area, breakpoint sites were distributed more evenly (*p* = 2.24e-02; see Fig. 1C).

**Fig. 1:**
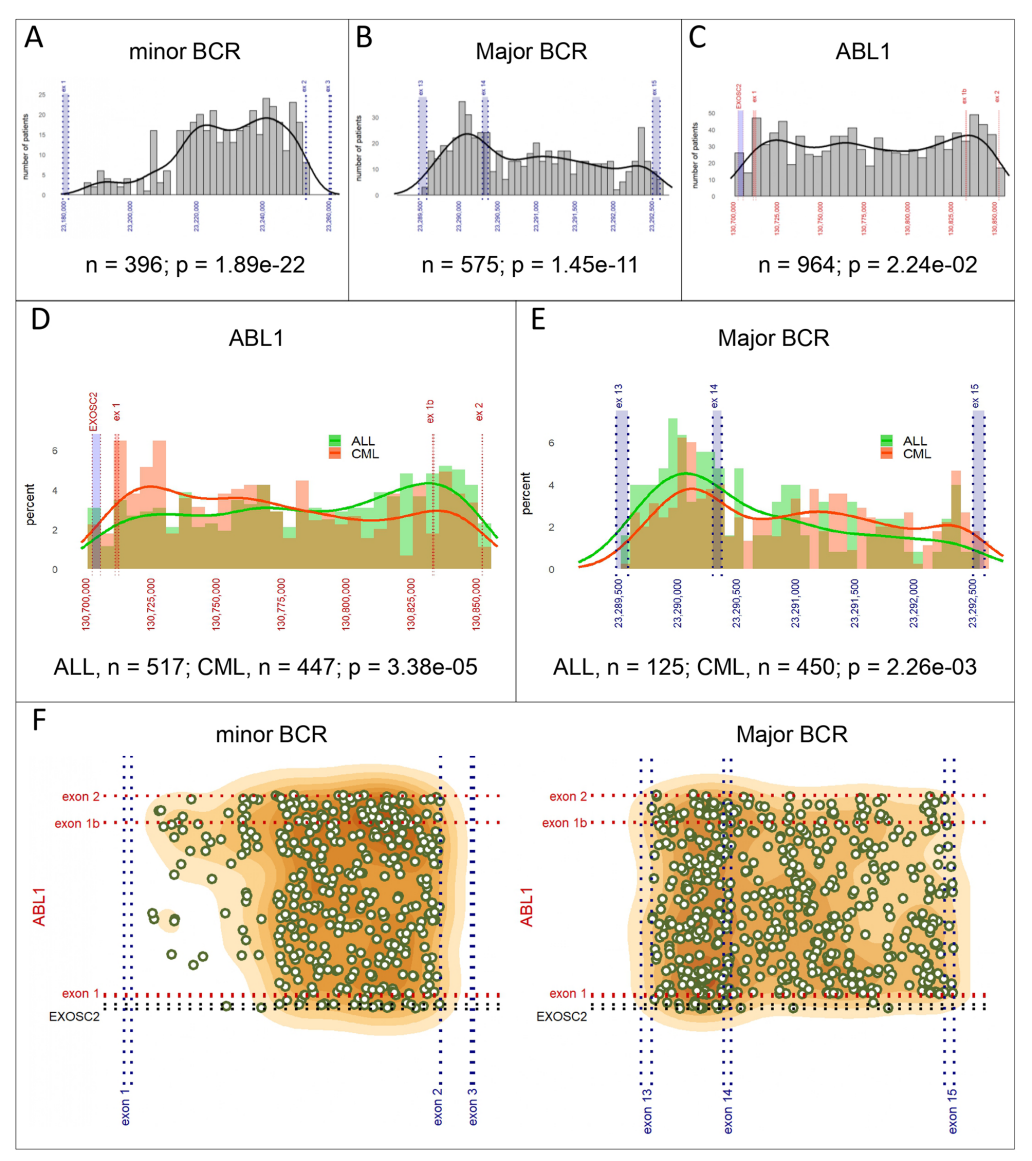
*BCR* and *ABL1* breakpoint distribution. Breakpoint distribution within the minor *BCR* **(A)**, Major *BCR* **(B)** and *ABL1* **(C)** breakpoint areas; comparison of breakpoints distribution between ALL and CML patients within *ABL1* **(D)** and Major *BCR* **(E)** areas; overall distribution of breakpoints and the relationship between breakpoint location on the *ABL1* side and in the minor and Major *BCR* regions. Gene coordinates are given according to GRCh38/hg38. The uniformity of breakpoint site distribution was tested using Pearson’s Chi-Squared test, comparison of breakpoint distribution between the groups was tested using Kolmogorov-Smirnov test. Images adapted from the “Break-App” web tool

Importantly, the breakpoint distribution within the *ABL1* gene differed significantly between CML and ALL patients (p = 3.38e-05; see Fig. 1D), with a higher accumulation of breakpoints in the 5’ part of the *ABL1* breakpoint area in CML and in the 3’ part of the area in ALL. This difference in breakpoint distribution was not driven by the type of fusion (minor vs. Major *BCR*) as the difference between CML and ALL was still apparent when only Major *BCR::ABL1*-positive patients were analyzed (445 CML and 123 ALL; *p* = 2.11e-02). Moreover, the different breakpoint distribution between CML and ALL patients was also evident in the Major *BCR* area (450 CML and 125 ALL; *p* = 2.26e-03, see Fig. 1E).

Using logistic regression, several tests were performed to evaluate the influence of sex, age and/or type of fusion (minor vs. Major *BCR* fusion [for *ABL1* breakpoints in ALL patients]). Only age at diagnosis was found to moderately influence the breakpoint distribution within the minor *BCR* area in ALL (≤ 16 [*n* = 266] vs. > 16 [*n* = 127] years; *p* = 7.89e-03; see Additional Fig. 1). The overall distribution of the breakpoints is shown in Fig. 1F.

The detailed analysis of genomic breakpoints showed that fusions are mostly formed in loci with short homologies (48.6%; median length = 1 bp, range 1–71 bp), by blunt-end junctions (36.6%) or by a junction with the insertion of a few random nucleotides (12.4%; 1–42 bp, median length = 2.5 bp; see Fig. 2A). This primary structure suggests that non-homologous end joining (NHEJ) is responsible for the double-strand break repair, consistent with other findings [1, 4–6]. However, in some cases, *BCR* and *ABL1* gene fusion may be a more complex process, as evidenced by insertions of part of chromosome 9 (including the *ABL1* gene) into the *BCR* or insertions of sequences (up to 12.3 kbp) from another chromosome between *BCR* and *ABL1*. For more details regarding these few specific exceptions see Additional Results.

**Fig. 2:**
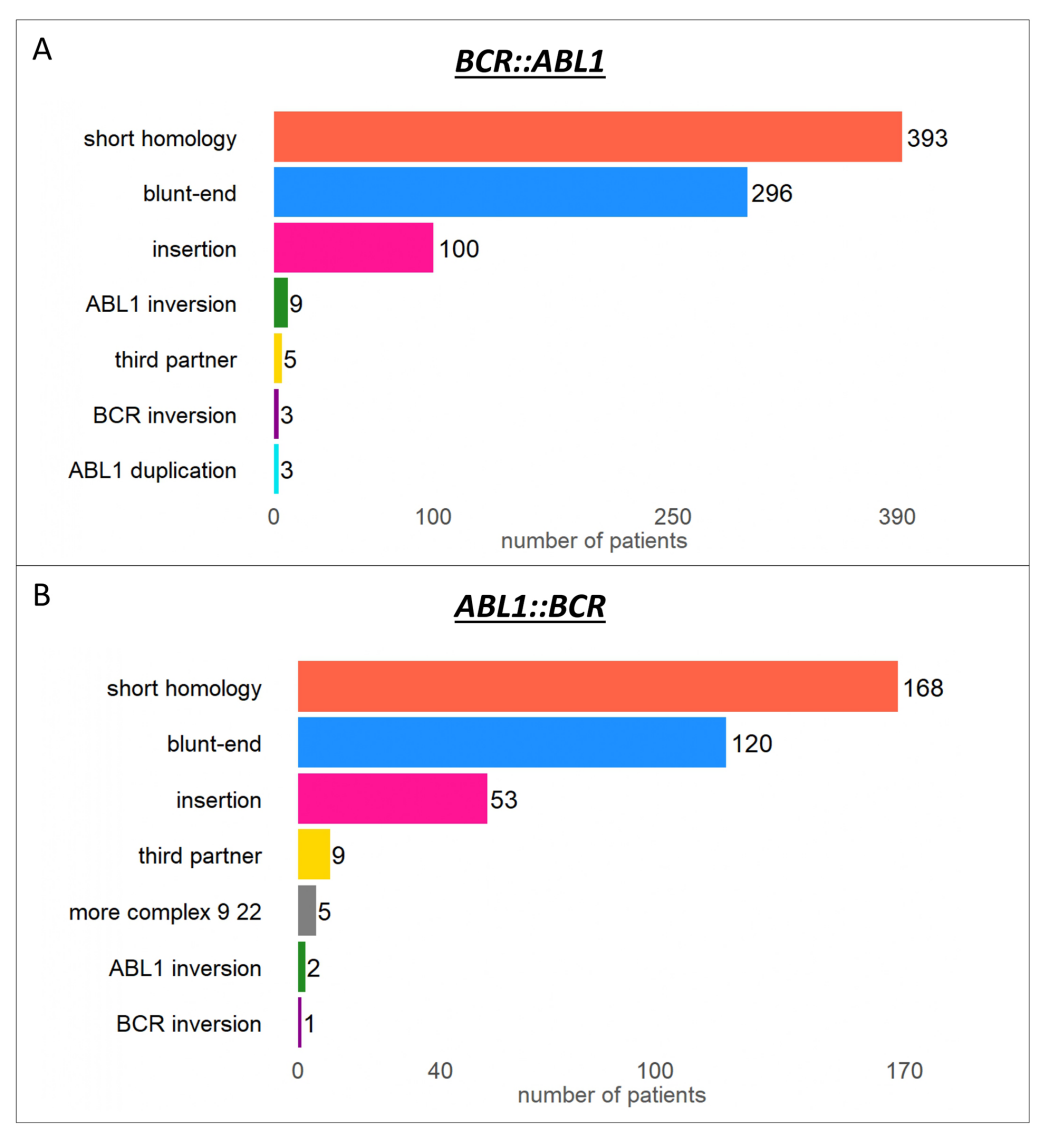
Primary structure of *BCR::ABL1* **(A)** and *ABL1::BCR* **(B)** breakpoints Images adapted from the “Break-App” web tool

We did not specifically look for the reciprocal *ABL1*::*BCR* fusion in all patients. However, the usage of NGS custom target enrichment enabled us to detect this breakpoint incidentally in some patients. In total, we characterized the *ABL1*::*BCR* junction in 415 (43%) patients (332 ALL, 83 CML) with a known *BCR*::*ABL1* sequence. The distribution of reciprocal breakpoints was highly consistent with the *BCR*::*ABL1* fusions, as the vast majority of the “forward” (*BCR*::*ABL1)* and “reciprocal” (*ABL1*::*BCR)* breakpoints in the given gene were located within a ± 100 bp window (81% in *BCR* and 80% in *ABL1*). In 42 patients (10%) the *BCR*::*ABL1* and *ABL1*::*BCR* breakpoints were located within a ± 1 bp on both fusion partners; in 14 patients, a perfectly balanced translocation was detected (see Additional Fig. 2). Thus, in majority of cases the t(9;22) translocation was perfectly reciprocal or very close to it even at the nucleotide level. Large deletions (over 10 kbp) of chromosomes 22 and/or 9 (which can be identified in cases with both *BCR*::*ABL1* and *ABL1*::*BCR* fusion available) were present relatively rarely (*n* = 20 [the largest deletion 2,171 kbp] and *n* = 21 [the largest deletion 1,005 kbp], respectively; i.e. 41/415 = 10% of patients). Duplications larger than 10 kbp were detected almost exclusively on chromosome 9 (*n* = 7; the largest duplication ~ 2,900 kbp; chromosome 22: *n* = 1; ~ 55 kbp). This analysis may be slightly biased as we did not specifically look for the reciprocal fusion in all patients and in some cases where the reciprocal fusion was not retrieved directly in the target enrichment NGS output, larger structural aberrations may have been involved, resulting in possible under-representation of more complex translocations. However, this bias would not dramatically impact our findings, since in a consecutive series of 404 patients analyzed by the same NGS approach, the reciprocal fusion was included in the NGS output in 87% of cases; the remaining 13% might comprise some further cases with more complex mechanism of fusion.

The primary structure of *ABL1*::*BCR* fusions corresponded to that of *BCR*::*ABL1* counterparts, with the vast majority of fusion sequences involving short homologies, short insertions and blunt-end junctions (Fig. 2B). The overall comparison of *BCR*::*ABL1* and *ABL1*::*BCR* fusions is listed in Additional Table 2.

We searched for RSS, specific motifs known to mediate DNA breaks (59 motifs, adopted from Ross et al., 2013) [4] and interspersed and other types of repeats within particular DNA areas (see Additional Methods). Despite the non-random pattern in both *BCR* regions, we did not find any significant association between the localization of breakpoints and any type of DNA motif or DNA sequences with specific chromatin structure or any evidence of breakpoint clustering within or in the neighborhood (± 10 bp) of any searched DNA or epigenetic motif. For further details see Additional Table 3 and Additional Results.

In our previous studies on *BCR::ABL1*-positive leukemias [7, 10–12], we defined “CML-like” leukemias, diagnosed as ALL but exhibiting *BCR::ABL1* fusion in multipotent, not fully leukemic progenitors, biologically resembling CML in lymphoid blast crisis. It would be intriguing to compare whether the distribution of fusions in these CML-like leukemias differs from typical ALL and is closer to that of typical CML. Our data indeed suggest slightly more frequent breaks in the 5’ portion of *ABL1* and downstream of intron 13 of *BCR* (more typical for CML) in CML-like leukemias than in typical ALL (data not shown), but the differences are relatively small and do not reach statistical significance, possibly due to the limited number of patients for this analysis (99 typical ALL vs. 53 CML-like leukemias).

To visualize our results and enable data browsing and various analyses of *BCR*::*ABL1* breakpoint positions, we developed an interactive “Break-App” web tool (available at https://clip.lf2.cuni.cz/break-app). The web tool enables the analysis of breakpoint sites distribution in general or within/between particular subgroups (diagnosis, sex, age-specific, etc.). Furthermore, it comprises detailed information about (i) the primary structure of the breakpoints, (ii) *ABL1*::*BCR* breakpoints, (iii) breakpoint positions with respect to DNA motifs and chromatin structure; (iv) detailed information about *KMT2A* breakpoints (see Additional Results). We plan to update this tool regularly with new data. Not only can further *BCR::ABL1* breakpoint positions be easily included, but, if desirable, the tool can also be adapted for other fusions or breakpoints.

Characterizing primary aberrations, including gene fusions, at the DNA level aids in understanding their origin and mechanisms, with practical applications such as obtaining patient-specific, highly sensitive targets for detecting residual leukemic cells [7, 11–15]. Advances in molecular techniques, particularly massively parallel sequencing, have significantly enhanced the feasibility of detecting genomic fusions, enabling efficient identification of breakpoint sites in a shorter time, regardless of the length and complexity of breakpoint regions in fusion partners. Previous publications on *BCR::ABL1*-positive leukemia have primarily focused on the Major form of the translocation due to technical challenges in determining breakpoints in the substantially larger minor *BCR* region (~ 2.9 vs. ~71.5 kbp).

In conclusion, our study of *BCR*::*ABL1* fusions is based on the largest and most complex cohort of patients with *BCR::ABL1* fusion identified at the DNA level to date. The complexity of our cohort allowed for the first time comparison of breakpoint distribution in CML vs. *BCR*::*ABL1*-positive ALL, revealing significant differences in both *ABL1* and Major *BCR* loci. Importantly, our data are not biased towards only ‘canonical’ fusions, as the NGS approach allowed us to characterize genomic breakpoints in all patients where at least one side of the fusion is located in the area covered by our probes. No DNA or epigenetic motif responsible for the non-random distribution was found. Taken together, our data suggest that physical colocalization and chromatin accessibility, which change with the developmental stage of the cell (hence the difference between ALL that arises in a committed lymphoid progenitor, and CML that arises in a stem/multipotent cell), are more critical factors influencing breakpoint localization than the presence of specific DNA motifs. While we offer here a detailed insight and analysis of the genomic breakpoints, the cause and exact molecular mechanism underlying the origin of double-strand breaks in *BCR* and *ABL1* genes and their fusion remains to be resolved.

### Electronic supplementary material

Below is the link to the electronic supplementary material.

Supplementary Material 1

Supplementary Material 2

## Data Availability

The datasets generated during and/or analyzed during the current study are included in this published article and its Additional files and/or are available from the corresponding author on reasonable request.

## Abbreviations

ALL: Acute lymphoblastic leukemia
CML: Chronic myeloid leukemia
NGS: Next generation sequencing
NHEJ: Non–homologous end joining
PCR: Polymerase chain reaction
