# Supplementary material for: Distinct pattern of genomic breakpoints in CML and BCR::ABL1-positive ALL: analysis of 971 patients
Source: Mol Cancer. 2024 Jul 5;23:138. doi: 10.1186/s12943-024-02053-4 (PMC11229488; doi:10.1186/s12943-024-02053-4)

# **Distinct pattern of genomic breakpoints in CML and *BCR::ABL1*-positive ALL: Analysis of 971 patients**

Lenka Hovorkova, Lucie Winkowska, Justina Skorepova, Manuela Krumbholz, Adela Benesova, Vaclava Polivkova, Julia Alten, Michela Bardini, Claus Meyer, Rathana Kim, Toby N Trahair, Emmanuelle Clappier, Sabina Chiaretti, Michelle Henderson, Rosemary Sutton, Lucie Sramkova, Jan Stary, Katerina Machova Polakova, Rolf Marschalek, Markus Metzler, Giovanni Cazzaniga, Gunnar Cario, Jan Trka, Marketa Zaliova and Jan Zuna

## **ADDITIONAL DATA**

## **Additional Methods**

*Patients & Breakpoint detection*

BCR::ABL1 genomic breakpoints were characterized by multiplex long-distance PCR (1) followed by Sanger sequencing or sequencing on the GS Junior platform (454 next-generation sequencing technology, Roche Diagnostics, CA, USA) (2); or using NGS Custom Target Enrichment (SS QXT Reagent Kit and SS XT HS2 Reagent Kit; Agilent, CA, USA) or Nextera® Rapid Capture Custom Enrichment protocol (Illumina, CA, USA), followed by sequencing on MiSeq or NextSeq 550 (Illumina, CA, USA).(3) Data from target enrichment sequencing were analyzed using NextGene (SoftGenetics, PA, USA), STAR(4) and deFuse(5) or Cicero(6) and manually curated.

Fusion sequences of primary (n = 1427) and secondary (n = 61) leukemias with *KMT2A* gene rearrangement (partly previously published (7)) were obtained from the Diagnostic Center of Acute Leukemia (DCAL) in Frankfurt, Germany, and were used as a validation set of samples to verify the ability to identify potential hot-spots of genomic breakpoints.

The project was approved by the Institutional Review Board of University Hospital Motol (Czech Republic) and Hunter Human Research Ethics Committee HNE 2019/ETH01219 (multisite, Australia). Informed consent was obtained following the Declaration of Helsinki.

*Analysis tools*

Alignment against GRCh 38 was performed using one of the following tools: BLAST, ENSEMBL or BLAT (University of California Santa Cruz). For further analyses and visualization (including comparison of breakpoint distribution in various subgroups with statistical calculation; comparison, visualization and analysis of primary breakpoint structure; visualization of *BCR::ABL1* vs. *ABL1::BCR* breakpoints and its structure; analysis of colocalization of breakpoints with DNA motifs and epigenetic features), the following R (v. 4.1.2)(8) tools and packages were used: R Studio (v. 2021.09.1.372)(9), BSgenome.Hsapiens.UCSC.hg38 (v. 1.4.4)(10), DT (v. 0.22)(11), gridExtra (v. 2.3)(12), gviz (v. 1.38.4)(13), ggplot2 (v. 3.3.5)(14), htmltools (v. 0.5.2)(15), plyranges (v. 1.14.0)(16), RCurl (v. 1.98.-1.6)(17), reshape2 (v. 1.4.4)(18), rtracklayer (v. 1.54.0)(19), shiny (v. 1.7.1)(20), shinyauthr (v. 1.0.0)(21), shinyjs (v. 2.0.0)(22), shinythemes (v. 1.2.0)(23), spgs (v. 1.0-3)(24), TxDb.Hsapiens.UCSC.hg38.knownGene_(v. 3.14.0; the database defined intron/exon boundaries for further analysis)(25).

*Statistic tests*

The uniformity of breakpoint site distribution within particular gene area was tested using Pearson's Chi-Squared test. Kolmogorov-Smirnov test was used to compare breakpoint positions between two groups of patients. Logistic regression was used to test the effects of particular variables to the probability of the breakpoint distribution.

*Motif search and epigenetic data*

The RSS database (26), MEME software (27) and RepeatMasker (28) were used to search for RSS, specific motifs known to mediate DNA breaks (59 motifs, adopted from Ross et al.,

2013)(29) and interspersed and other types of repeats within particular DNA areas (breakpoint regions of *BCR*, *ABL1* and *KMT2A* genes), respectively. Data regarding DNA accessibility (ATAC-seq, ChIA-PET, ChIP-seq, DNase-seq, WGB) in K562 cell line and particular cell types possibly involved in breakpoint origin were downloaded from ENCODE (30, 31), McGill Epigenomics Mapping Centre (32), and studies published elsewhere.(33-35)

## Additional Results

*Primary structure of breakpoints*

The detailed analysis of genomic breakpoints showed that fusions are mostly formed in loci with short homologies (48.6 %; median length = 1 bp, range 1 – 71 bp), by blunt-end junctions (36.6 %) or by a junction with the insertion of a few random nucleotides (12.4 %; 1 – 42 bp, median length = 2.5 bp; see the main text). However, several atypical fusions were detected – i) *BCR::*[inverted *ABL1* segment]::*ABL1* (n = 9; median length of inverted segment = 35 bp; notably, in 7/8 such patients with Major-*BCR::ABL1* fusion, the breakpoint occurred within 1 kbp area at the *BCR* side), ii) *BCR*::[third partner]::*ABL1* (n = 5; insertion length 935 – 12,300 bp; the third partners were from chromosomes 3 [*IGSF11* gene], 4 [*INPP4B*], 5 [*SRD5A1*], 12 [intergenic region] and 17 [*ASIC2*]), iii) *BCR*::[inverted *BCR* segment]::*ABL1* (n = 3; length of inverted segment 76 – 270 bp), iv) *BCR*::[duplicated *ABL1* segment]::*ABL1* (n = 3; length of duplicated segment 11 – 86 bp). In two patients, the insertion of DNA from chromosome 9 (*ABL1* and downstream sequence in total length of ~ 97 kbp and ~ 190 kbp) into the *BCR* gene was detected.

*Association of breakpoints with DNA motifs and chromatin structure*

We did not find any significant association between the localization of breakpoints and any type of DNA motif or DNA sequences with specific chromatin structure (see the main text).
Generally, breakpoints in specific motifs (RSS, SINE/Alu, LINE/L1) on both fusion partners were rare. Regarding RSS motifs, taking into account the 12/23 spacer rule and RSS sequence orientation (+/-), a breakpoint possibly produced by RSS mechanism was found in only 3 patients. Using MEME software (searching for selected DNA motifs, see Additional Table 3),

we found breakpoints localized within the same DNA motif family on both *BCR* and *ABL1* sides in seven patients – 1x XY32 homopurine pyrimidine H palindrome motif (minor *BCR::ABL1*), 6x human minisatellite (1x minor, 5x Major *BCR::ABL1*). When analyzing DNA motifs defined by RepeatMasker software, the breakpoints in the same motif family (or its fragment) were found in 44 (4.5%) patients (25x minor, 19x Major *BCR::ABL1*; 15x Line/L1, 28x SINE/Alu and 1x SINE/MIR). Of those, in all six patients, in whom larger homologies (22 – 71 bp) were detected in the *BCR*::*ABL1* fusions (all minor *BCR*::*ABL1*), and in one patient with translocation including insertion from a third partner from chromosome 17 (4,760 bp), the breakpoints were located in SINE/Alu repeats on both *BCR* and *ABL1* sides. Moreover, the breakpoints in three of the patients with large homologies (2x 71 bp and 1x 40 bp) occurred within exactly the same specific SINE/Alu repeat on the *BCR* side. Although these cases are noteworthy and may represent one of the mechanisms of breakpoints origin, they represent a subtle minority in the entire cohort of almost one thousand of analyzed patients. The overall overview of the DNA motifs is shown in Additional Table 3.

*Secondary leukemias with KMT2A rearrangement*

To validate our approach of mapping breakpoints to DNA/epigenetic motifs, we complemented our cohort with 1488 leukemia patients with KMT2A rearrangements, including 61 patients with secondary leukemia. Using the “Break-App” web tool, a breakpoint hotspot near KMT2A exon 12, in close proximity to the Topoisomerase II consensus cleavage site, DNAse I HS and CTCF-binding site (36) was clearly apparent and visible, particularly in secondary leukemias harboring the KMT2A rearrangement (Additional Figure 3), confirming effectiveness of our approach.

**References to Additional Data**


1. Hovorkova L, Zaliova M, Venn NC, Bleckmann K, Trkova M, Potuckova E, et al. Monitoring of childhood ALL using BCR-ABL1 genomic breakpoints identifies a subgroup with CML-like biology. Blood. 2017;129(20):2771-81.
2. Linhartova J, Hovorkova L, Soverini S, Benesova A, Jaruskova M, Klamova H, et al. Characterization of 46 patient-specific BCR-ABL1 fusions and detection of SNPs upstream and downstream the breakpoints in chronic myeloid leukemia using next generation sequencing. Mol Cancer. 2015;14:89.
3. Zuna J, Hovorkova L, Krotka J, Koehrmann A, Bardini M, Winkowska L, et al. Minimal residual disease in BCR::ABL1-positive acute lymphoblastic leukemia: different significance in typical ALL and in CML-like disease. Leukemia. 2022;36(12):2793-801.
4. Dobin A, Davis CA, Schlesinger F, Drenkow J, Zaleski C, Jha S, et al. STAR: ultrafast universal RNA-seq aligner. Bioinformatics. 2013;29(1):15-21.
5. McPherson A, Hormozdiari F, Zayed A, Giuliany R, Ha G, Sun MG, et al. deFuse: an algorithm for gene fusion discovery in tumor RNA-Seq data. PLoS Comput Biol. 2011;7(5):19.
6. Tian L, Li Y, Edmonson MN, Zhou X, Newman S, McLeod C, et al. CICERO: a versatile method for detecting complex and diverse driver fusions using cancer RNA sequencing data. Genome Biol. 2020;21(1):126.
7. Meyer C, Larghero P, Almeida Lopes B, Burmeister T, Groger D, Sutton R, et al. The KMT2A recombinome of acute leukemias in 2023. Leukemia. 2023;37(5):988-1005.
8. R Core Team. R: A language and environment for statistical computing. 2021.
9. RStudio Team. RStudio: Integrated Development Environment for R. 2015.
10. The Bioconductor Dev Team. BSgenome.Hsapiens.UCSC.hg38: Full genome sequences for Homo sapiens (UCSC version hg38, based on GRCh38.p13). R package version 1.4.4. 2021.
11. Xie Y, Cheng J, Tan X. DT: A Wrapper of the JavaScript Library 'DataTables'. 2022.
12. Auguie B. gridExtra: Miscellaneous Functions for "Grid" Graphics. 2017.
13. Hahne F, Ivanek R. Visualizing Genomic Data Using Gviz and Bioconductor. Methods Mol Biol. 2016;1418:335-51.

14. Wickham H. ggplot2: Elegant Graphics for Data Analysis.: Springer-Verlag New York; 2016.
15. Cheng J, Sievert C, Schloerke B, Chang W, Xie Y, Allen J. htmltools: Tools for HTML. 2022.
16. Lee S, Cook D, Lawrence M. plyranges: a grammar of genomic data transformation. Genome Biology. 2019;20(1):4.
17. Lang DT. RCurl: General Network (HTTP/FTP/...) Client Interface for R. 2021.
18. Wickham H. Reshaping Data with the {reshape} Package. Journal of Statistical Software. 2007;21(12):1-20.
19. Lawrence M, Gentleman R, Carey V. rtracklayer: an R package for interfacing with genome browsers. Bioinformatics. 2009;25(14):1841-2.
20. Chang W, Cheng J, Allaire JJ, Sievert C, Schloerke B, Xie Y, et al. shiny: Web Application Framework for R. R package version 1.7.2. 2022.
21. Campbell P. shinyauthr: 'Shiny' Authentication Modules. 2021.
22. Attali D. shinyjs: Easily Improve the User Experience of Your Shiny Apps in Seconds. 2021.
23. Chang W. shinythemes: Themes for Shiny. 2021.
24. Hart A, Martínez S. spgs: Statistical Patterns in Genomic Sequences. 2019.
25. Bioconductor Core Team and Bioconductor Package Maintainer. TxDb.Hsapiens.UCSC.hg38.knownGene: Annotation package for TxDb object(s). 2021.
26. Merelli I, Guffanti A, Fabbri M, Cocito A, Furia L, Grazini U, et al. RSSsite: a reference database and prediction tool for the identification of cryptic Recombination Signal Sequences in human and murine genomes. Nucleic Acids Res. 2010;38(Web Server issue):W262-7.
27. Bailey TL, Johnson J, Grant CE, Noble WS. The MEME Suite. Nucleic Acids Res. 2015;43(W1):W39-49.
28. Smit A, Hubley R, Green P. RepeatMasker Open-4.0. 2013-2015 [Available from: http://www.repeatmasker.org.
29. Ross DM, O'Hely M, Bartley PA, Dang P, Score J, Goyne JM, et al. Distribution of genomic breakpoints in chronic myeloid leukemia: analysis of 308 patients. Leukemia. 2013;27(10):2105-7.
30. An integrated encyclopedia of DNA elements in the human genome. Nature. 2012;489(7414):57-74.

31. Luo Y, Hitz BC, Gabdank I, Hilton JA, Kagda MS, Lam B, et al. New developments on the Encyclopedia of DNA Elements (ENCODE) data portal. Nucleic Acids Res. 2020;48(D1):D882-D9.
32. Bujold D, Morais DAL, Gauthier C, Cote C, Caron M, Kwan T, et al. The International Human Epigenome Consortium Data Portal. Cell Syst. 2016;3(5):496-9 e2.
33. Corces MR, Buenrostro JD, Wu B, Greenside PG, Chan SM, Koenig JL, et al. Lineage-specific and single-cell chromatin accessibility charts human hematopoiesis and leukemia evolution. Nat Genet. 2016;48(10):1193-203.
34. Satpathy AT, Granja JM, Yost KE, Qi Y, Meschi F, McDermott GP, et al. Massively parallel single-cell chromatin landscapes of human immune cell development and intratumoral T cell exhaustion. Nat Biotechnol. 2019;37(8):925-36.
35. Takayama N, Murison A, Takayanagi SI, Arlidge C, Zhou S, Garcia-Prat L, et al. The Transition from Quiescent to Activated States in Human Hematopoietic Stem Cells Is Governed by Dynamic 3D Genome Reorganization. Cell Stem Cell. 2021;28(3):488-501 e10.
36. Cowell IG, Austin CA. DNA fragility at the KMT2A/MLL locus: insights from old and new technologies. Open Biol. 2023;13(1):220232.

## Additional Table legends

For Additional Tables see file Additional Tables.xls

*Additional Table 1:*

**Breakpoint positions and basic characteristics of the patients**

*Additional Table 2:*

**Structure of breakpoints - comparison of BCR::ABL1 and ABL1::BCR fusions**

*Additional Table 3:*

**Summary of DNA motifs and their association with breakpoints**

The table shows the number of occurrences of each motif within the respective breakpoint region and the total proportion of the breakpoint region occupied by these motifs (percentage shown for motif alone and for motif +/- 10 bp surrounding sequence). Number and percentage of patients with a breakpoint within the motif (and also within +/- 10 bp surrounding sequence) is shown. Relative difference between the percentage of sequence occupied by the motif and percentage of patients with a breakpoint within the motif (and also +/- 10 bp surrounding sequence) is also shown (hence values near zero suggesting random association, positive values enrichment and negative values reduction of breakpoints within the particular motif). Relative differences ≥ 10% are highlighted in colours (+10 to +30 % in yellow; +31 to +50 % in green; > +50% in red) when breakpoints colocalise with motifs in > 10 patients.

## Additional Figures

*Additional Figure 1:*

**Breakpoint distribution of younger (≤ 16 years) and older (> 16 years) patients within minor BCR.**

Gene coordinates are given according to GRCh38/hg38. Comparison of breakpoint distribution between the groups was tested using Kolmogorov-Smirnov test. Images adapted from the "Break-App" web tool.

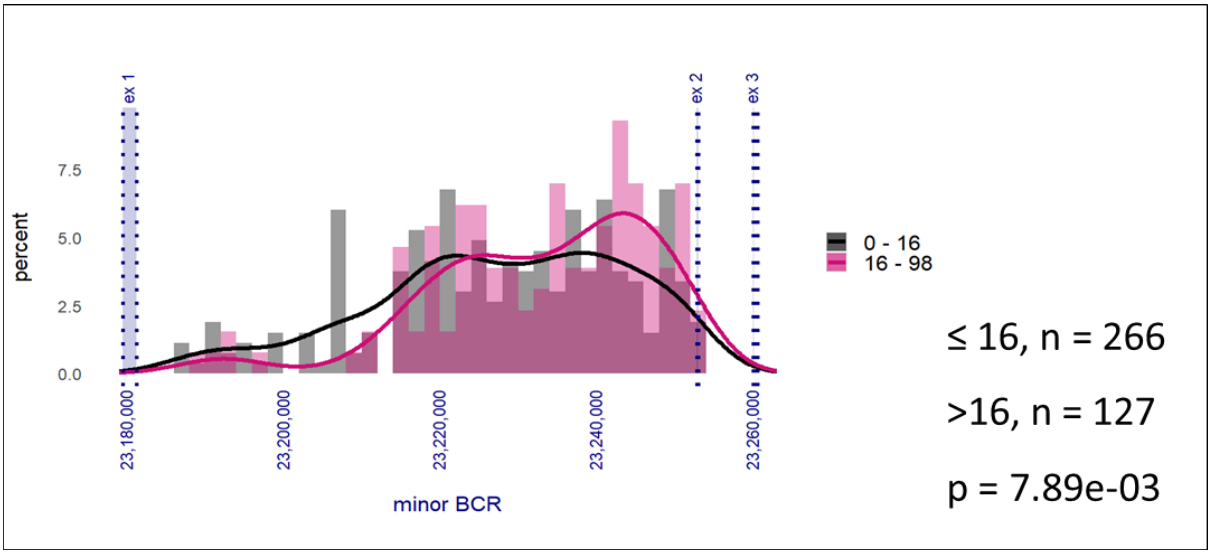


*Additional Figure 2*:

**Projection of BCR::ABL1 and reciprocal ABL1::BCR fusions in 415 patients.**

Each patient is represented by a point. If 2 or more patients overlap at one point, this is represented by a larger point size (see caption below). Positive values show duplication, negative values deletion of BCR or ABL1 DNA at the breakpoints; 14 patients at coordinates 0:0 represent perfectly reciprocal fusions. Three graphs (zoomed at ±1000 bp (A), ±100 bp (B) and ±25 bp (C) area around a theoretically perfectly reciprocal fusion) show 301, 253 and 219 patients, respectively. Primary structure of the involved breakpoints is shown in attached tables. Images adapted from the "Break-App" web tool.

A

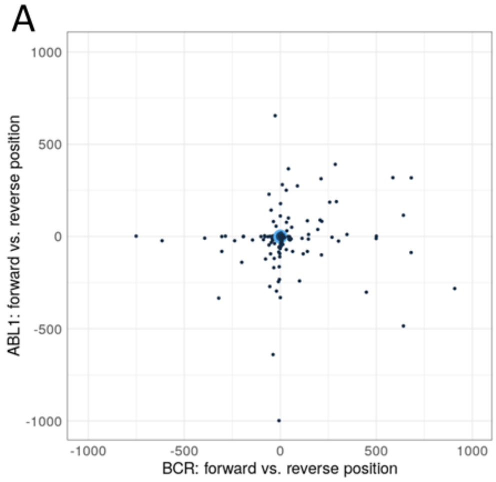


| | TYPE | BCR::ABL1 | | | |
|---|---|---|---|---|---|
| | | Short homology | Blunt-end | Insertion | Other |
| ABL1::BCR | Short homology | 88 | 42 | 13 | 1 |
| | Blunt-end | 49 | 46 | 8 | 1 |
| | Insertion | 15 | 8 | 20 | |
| | Other | 2 | 4 | 3 | 1 |

n = 301

B

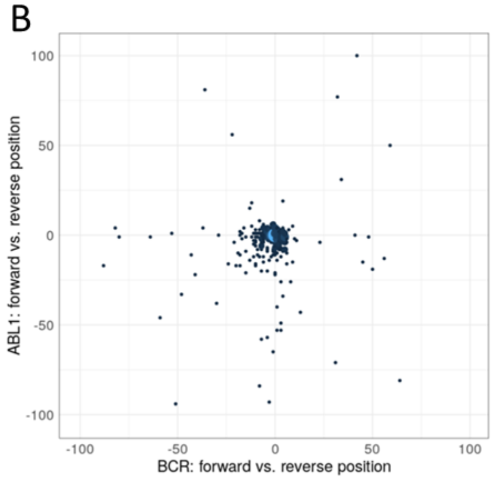


| | TYPE | BCR::ABL1 | | | |
|---|---|---|---|---|---|
| | | Short homology | Blunt-end | Insertion | Other |
| ABL1::BCR | Short homology | 76 | 29 | 11 | |
| | Blunt-end | 42 | 40 | 7 | 1 |
| | Insertion | 14 | 7 | 19 | |
| | Other | 2 | 4 | 1 | |

n = 253

C

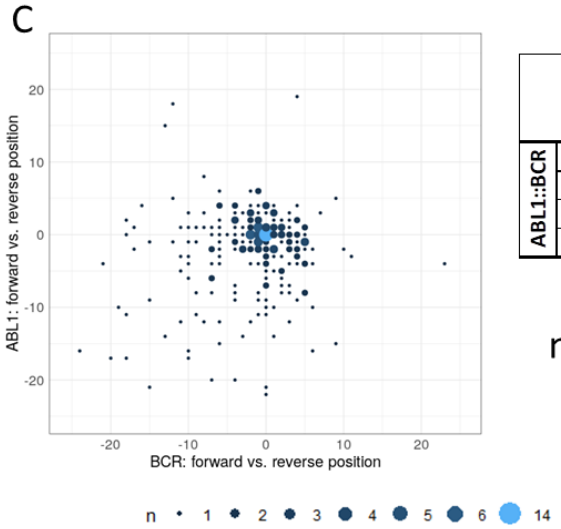


| | TYPE | BCR::ABL1 | | | |
|---|---|---|---|---|---|
| | | Short homology | Blunt-end | Insertion | Other |
| ABL1::BCR | Short homology | 66 | 25 | 10 | |
| | Blunt-end | 33 | 38 | 7 | |
| | Insertion | 12 | 7 | 14 | |
| | Other | 2 | 4 | 1 | |

n = 219

*Additional Figure 3*:

**Distribution of breakpoints within KMT2A gene in 61 patients with secondary leukemia.**

Scheme of KMT2A gene breakpoint cluster region between exons 8 and 15 showing a breakpoint hotspot near exon 12, in close proximity to the DNAse I HS and CTCF-binding site (ChiP-seq, DNAseseq) and Topoisomerase II consensus cleavage site colocalization. Image adapted from the “Break-App” web tool.

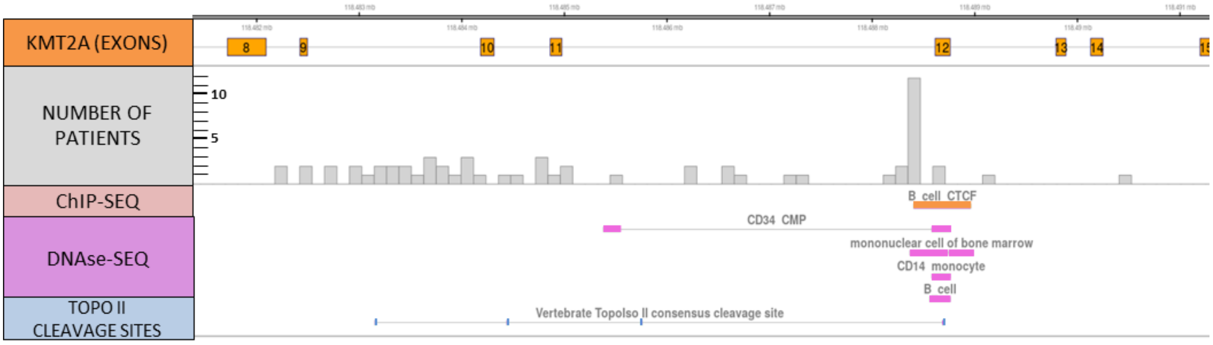

Supplement: Supplementary file 2 — Supplementary Material 2 [file 12943_2024_2053_MOESM2_ESM.pdf]
